# Recent Advances in 3D Food Printing: A Review with Focus on Personalized Nutrition and Functional Food Applications

**DOI:** 10.17113/ftb.63.04.25.8946

**Published:** 2025-12-26

**Authors:** Rasheeda Meembidi, Mohan Chitradurga Obaiah, Remya Sasikala, Bindu Jaganath

**Affiliations:** 1 Fish Processing Division, ICAR-Central Institute of Fisheries Technology, CIFT Junction, Matsyapuri P. O., Kochi, 682029 Kerala, India; 2 Department of Food Science and Technology, FOST, KUFOS, Panangad Road, Madavana Junction, Kochi, 682506 Kerala, India

**Keywords:** food printing, extrusion, 3D designs, personalized nutrition, nutrient-rich products

## Abstract

The 3D printing technique offers immense opportunities to manufacture foods tailored to individual preferences, with added benefits to address malnutrition. Malnutrition is a major public health issue that impedes the development of nations worldwide. Fortification with functional ingredients is a promising approach to combat this problem. However, consumer acceptance of fortified foods remains low due to their bland taste and unfamiliar formats. This situation has created the demand for customized, fortified products made with novel technologies like 3D printing. This review compiles research findings from the last 15 years on 3D printing in food manufacturing. It provides a detailed review on various technological options available for 3D printing, with emphasis on recent research related to functional food. Previous studies have demonstrated that 3D food printing is a highly promising novel technology capable of providing personalized nutrition. 3D food printers primarily use extrusion technology to create nutrient-rich food products in various 3D designs, meeting both the aesthetic and functional preferences of consumers. This technology is expected to revolutionize the functional food industry in the near future, due to its various applications.

## INTRODUCTION

Three-dimensional (3D) printing, which emerged in the early 1980s, as a result of the fourth industrial revolution (*1*), has been gaining momentum in recent years. This technique has significant applications in sectors like medicine, aviation, textiles, civil engineering and the military (*2*). Raw materials like plastic and photopolymers were predominantly used for prototyping in 3D printing technology (*3*). The first 3D printer was constructed by 3D System in the 1990s (*4*). The ability to develop prototypes rapidly makes this technique unique compared to other printing methods. Three-dimensional printing of foods is the latest advancement of 3D printing, which has emerged only recently. The conceptual patterns of first-generation 3D food printers were presented to the public around 18 years ago. Researchers from the United Kingdom constructed the earliest version of a 3D printer for printing chocolates, which was considered the first model of a food printer. Subsequently, another 3D printer named ‘Foodini’ was launched by a Spanish company, which paved the way for the development of 3D printers. Since 2011, the 3D printing technique has been employed in the food industry (*5*). In 3D food printing, foods are fabricated additively in layers (*6*) from multiple ingredients, in complex designs chosen by the end user. This technique follows a clear sequential set of operations (*7*), which involves a digital process that initiates the development of a 3D CAD model for the preferred design. A slicing software is used to slice the models into their respective layers. During slicing, machine codes are generated for sliced layers, which can be transferred to the instrument to initiate printing of the selected recipe (*8*). A good synergy between the hardware and software of the printer system is essential for the efficient operation of a 3D food printer.

### Extrusion-based 3D printing

**Fig. 1:**
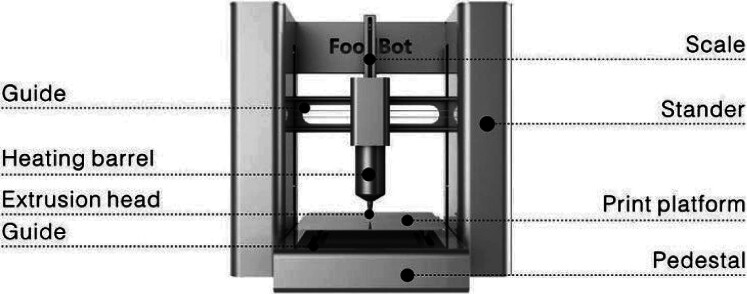
Schematic diagram of a FoodBot 3D food printer (scanned image from the manufacturer's manual)

**Fig. 2:**
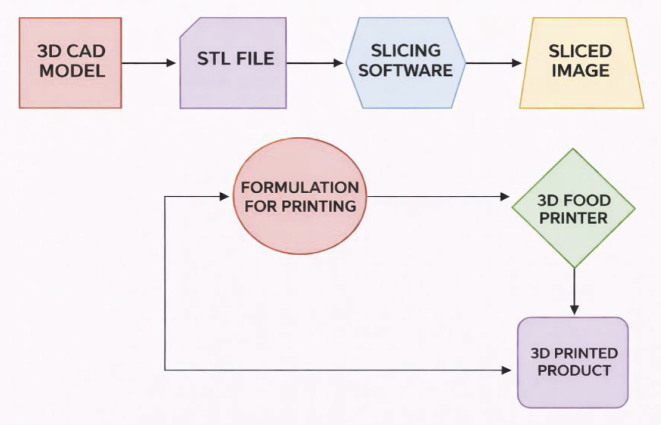
Schematic diagram of basic workflow of a 3D food printer

**Fig. 3:**
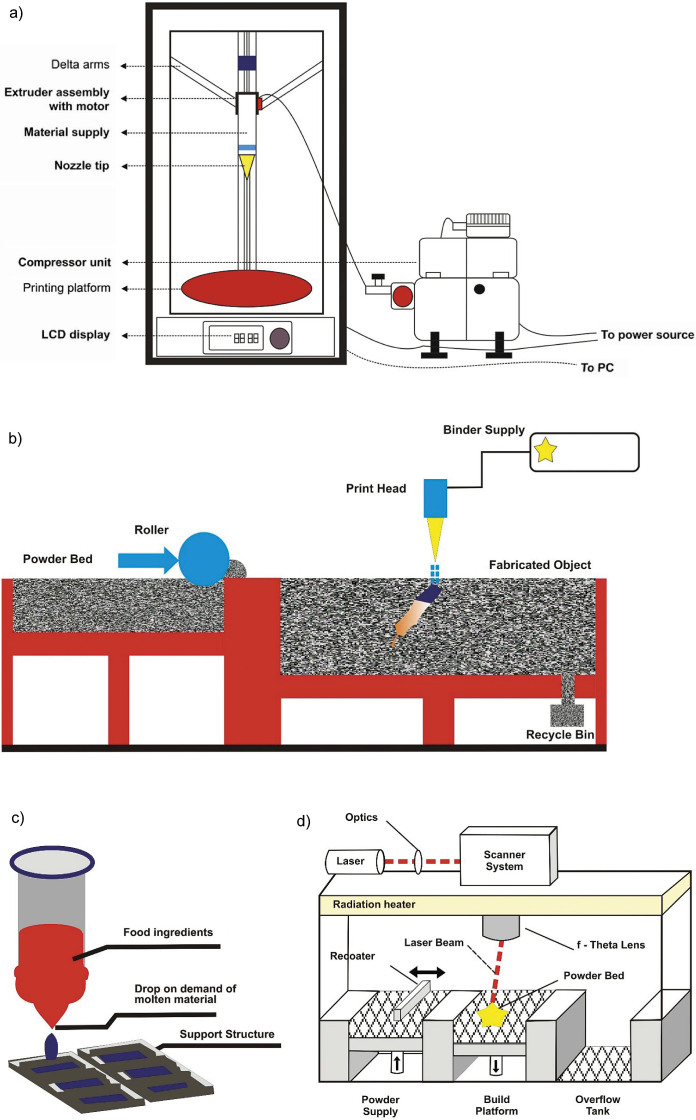
Schematic diagram of: a) extrusion-type 3D food printer, b) binder jet printer, c) inkjet printer, and d) selective laser sintering 3D printer (Corel Draw graphics editor)

As 3D food printing offers numerous benefits, various types of printers differing in their operating principles have been developed over the last few decades. A detailed description of each type is provided in the following section. Fig. 1 shows the schematic diagram of a FoodBot food printer. Fig. 2 gives the workflow of a 3D food printing machine. The four major techniques of food printing are extrusion-based printing, binder jet printing, inkjet printing and selective laser sintering. Among these technologies, the most commonly employed technique is extrusion printing (*9*). In this technique, food designs are formed through extrusion using a nozzle under static pressure. Both solid materials and low-viscosity pastes can be printed using this method. Food materials to be printed are loaded into the machine and then forced through the nozzle to form layers of food material, one over the other. Foods commonly printed by this method include dough, meat, paste and cheese (*10*). Fish surimi gel has been printed using an extrusion-type printer, and it was reported that the diameter and velocity of the nozzle, along with the extrusion rate, influence the quality of 3D printing (*11*). Different types of sugar cookies have been printed with an extrusion printer (*12*). Fig. 3a shows the schematic representation of an extrusion-oriented 3D printer. The major advantages of this technique are the low cost of basic models and ease of customization to accommodate a variety of raw materials. However, its disadvantages include low level of printing precision and a very long build time.

### Binder jet 3D printing

In the binder jet technique, layers of food-grade powders are spread uniformly over the fabrication platform (*13*). These layers are often sprayed with a stream of water to make them stable, minimizing the disturbances caused by binder jetting. Small binder droplets are dropped onto the surface of the powder bed from a printer head, binding the adjacent powder layers. The powder surface is then heated using a hot lamp to provide mechanical strength to withstand the printing process. These steps are performed sequentially for all layers (*13*). The properties of both the powder and the binder play a crucial role in efficient printing. This process has some benefits like faster fabrication, the ability to create complex structures, and reduced cost of ingredients. With this technology, Sugar Lab fabricated sculptural cakes with sugar and different flavour binders. Fig. 3b shows the schematic diagram of the binder jet printer. Binder jet 3D printing is a versatile technique and its production speed is greater than that of other techniques. Another advantage is the possibility of including support structures in the fabrication of layers. The disadvantage of this technique is the rough or grainy appearance of the end product. Producing fine and soft-textured products is very difficult with this technique. This type of printing requires post-processing step to reduce moisture content and improve the strength of the printed foods.

### Inkjet 3D printing

In the inkjet printing, food materials are dispensed from a heated head with multiple channels to the selected regions of the food surface (*14*). The droplets are dispensed in a drop-on-demand manner. They fall under gravity and are dried through solvent evaporation. These droplets can create two-and-a-half-dimensional images (*14*). This technique is commonly used for surface filling or decorating purposes. Baked items like cookies, cakes, pastries and pizza are decorated using this method (*15*). Food materials with complex structures are rarely printed by this method. Fig. 3c shows a schematic diagram of the inkjet printer. Inkjet printing offers advantages like high resolution of the prints and accurate printing. With this technique, a variety of raw materials can be used for 3D printing. The main disadvantage is the damage that can occur to the food after the processing due to its delicate structure.

### Selective laser sintering 3D printing

Selective laser sintering technology can be applied to powders, where they are sintered selectively to create any desired shapes (*16*). A sintering source like hot air or a laser is passed over a bed of powdered food spread evenly. The source moves across both x and y axes of the powder bed to bind the particles together. The sintering process is repeated continuously, so that the fused material attains a 3D geometry through simultaneous layering and binding of the particles (*16*). The food jetting printer from TNO (*17*) has used lasers for fusing sugars and Nesquik powders into 3D designs. The unsintered material is retained in place as a support for the whole structure. Candy Fab (*18*) used a stream of hot air at minimal speed to sinter a layer of sugar. Application of heat to the bed at a temperature lower than the melting point of the powdered material prevents thermal destruction of the product and enhances the binding of particles. Both hot air and laser sintering methods enable rapid fabrication of foods without requiring any post-printing operations. However, a limitation of this technique is that it can only sinter sugar and fat-containing particles with low melting points (*19*). Fig. 3d shows a schematic diagram of a selective laser sintering type 3D printer. The advantages and disadvantages of different printing techniques are summarised in Table 1 (*10*, *20*-*23*).

**Table 1:** Advantages and disadvantages of different printers

3D food printing technique	Advantage	Disadvantage
Extrusion- based	Initial low cost of the machines, availability of a wide range of ingredients, ease of customization (*10*)	Lower printing precision, longer fabrication time to manufacture sharp outer edges, anisotropic property of food after printing, difficulty in holding 3D structures during post printing (*10*)
Binder jetting	Shorter printing time (*20*), capability of fabricating foods with greater solid content, support materials incorporated automatically during layer formation, developing products from various materials, ease of colour printing on various food parts (*21*)	Limited to foods composed of sugar and starch powders (*21*), rough surface of the developed foods, necessitating post-printing treatments including temperature curing, lower nutritive value of the developed products (*22*)
Inkjet printing	Zero waste of model components, higher resolution and accuracy, possible use of diverse components and colours, higher fabrication speed (*10*)	Post-printing may destruct tiny and thin features, the support materials cannot be reused so it produces a lot of waste, simple design and used mainly for surface filling or decorative purposes (*10*)
Selective laser sintering	Complicated parts which cannot be fabricated by other methods can be created with ease, no need for external support, ideal for bulk production, highly accurate and precise (*23*)	The fabrication is expensive, needs a post-printing step, broader surfaces and small voids are challenging to fabricate with accuracy (*21*)

### Materials for 3D food printing

The materials used for printing are often classified into three main categories. Natively printable materials are those that can be extruded easily using syringes (*24*). Cake frosting, chocolate and cheese are some examples. These materials can be fully personalized regarding the nutritional, textural and sensory attributes. Some materials retain their shape for a long time after deposition, so post-processing is not necessary. Non-printable materials include food products that are consumed daily, such as meat, fish, rice, fruit and vegetables. They can be made printable only by incorporating compounds like hydrocolloids. Xanthan gum and gelatine are usually used for this purpose (*25*). The addition of suitable additives to these materials modifies their printing properties, resulting in a new formulation (*12*). Alternative ingredients include extracts from organisms such as algae, fungi, seaweed and lupins, along with insects as the newer sources of protein and fibre. According to the Insects Au Gratin Project, formulations containing insect powders, low-viscosity icings and cheese have been used as raw materials to fabricate foods and create tastier recipes (*26*). The leftovers from the current agriculture and food sector can be transformed into metabolites, enzymes and food-grade flavours, which are known for their biological activity. The advantages and disadvantages of the formulations used in printing are given in Table 2 (*11*, *27*-*30*).

**Table 2:** Advantages and disadvantages of different formulations used in 3D food printing

Type of formulation	Advantage	Disadvantage	Reference
Fruit- and vegetable-based	Sensory and antioxidant properties and phenolic content remain unchanged after printing, visually more appealing after printing	Difficulty in accurate replication of the computer-made design, especially for large scale applications; Sanitizing all the areas of the machine in contact with food was not easy	(*27*)
Egg-based	Printing precision was high, greater layer definition	Requires incorporation of filler like rice flour to enhance printability	(*28*)
Milk-based	Imitates high-protein food system, good similarity of the prints to the 3D designs	Not reported	(*29*)
Fish-based	Higher degree of resolution, similarity to the 3D model, less deformed	Requires addition of sodium chloride to reduce viscosity and improve printability	(*11*)
Cereal-based	Structural quality as well as nutritional profile was unaffected	Not reported	(*30*)

The key benefit of 3D food printing is the rapid fabrication of customized food products with complex geometric patterns and novel textures (*25*). As 3D foods are categorized as soft foods due to their high digestibility, they form an important part of geriatric nutrition, especially for the dysphagic community (*31*). Apart from designing foods with customized patterns, 3D printing develops foods with tailored nutritional content through the incorporation of multiple ingredients. The ability of food printing to provide personalized nutrition makes this technology an excellent solution for addressing nutritional disorders caused by deficiencies in different nutrients. This review aims to highlight the role of functional foods with bioactive ingredients in addressing nutrient deficiency disorders, which mainly prevail in developing nations like India, Nepal and various parts of Africa. The evolution of 3D food printers, numerous printing techniques employed and novel 3D-printed functional foods that could enhance and transform the realm of food, both aesthetically and functionally, are also thoroughly reviewed in this article.

## MALNUTRITION IN DEVELOPING COUNTRIES

Although malnourishment is recognized as a global challenge, Asia and Africa are the main sufferers of all forms of undernutrition (*32*). In India, a South Asian country, undernutrition is one of the main contributors to disease burden and a significant risk factor for increased mortality among children below the age of five (*33*). Although the GDP in India has increased by 50 %, one-quarter of the world’s undernourished children live in India (*34*). The most important reason is economic inequality, which leads to the consumption of diets lacking essential nutrients (*35*). According to the Ministry of Women and Child Development, approximately one million children in the country are deprived of proper nourishment. The disease burden caused by this condition, as well as the trends in various malnutrition indicators such as low birth mass, stunting and wasting in children, anaemia in children and women were evaluated in every state of India (*36*). These trends highlight the need to reduce the effects of these indicators significantly to achieve global 2030 targets, which in turn highlights the need for an integrated nutrition policy (*36*). In a study conducted in a rural village in South India, calcium and iron consumption were found to be low, especially among the people aged around 60 years (*37*). Around 96 % of women and 84 % of men were deficient in calcium. It was also observed that the diets of both men and women did not meet the recommended daily intake of iron. Deficiencies of calcium and iron can lead to disorders like anaemia and osteoporosis. About 26 % of the people had a low body mass index (BMI). That study highlighted the importance of community-based nutritional interventions (*37*). In another study conducted among students from Ernakulam district, anaemia was found to be prevalent in children from classes 6 to 9 (aged 11-14) (*38*). Iron deficiency-related anaemia remains one of the main health issues, among both children and teenagers in India. The prevalence of anaemia was higher in adolescent girls, and the intake of iron-rich diet was reported to be lower among the children (*38*).

In Nepal, malnourishment is a major health hazard among children under the age of five, particularly in rural areas (*39*). Undernutrition is also an important health concern in Africa, especially in the sub-Saharan region (*40*). Malnutrition in Africa poses a substantial public health and socioeconomic burden, requiring urgent attention and intervention (*41*). In 2020, about 264.2 million people in the sub-Saharan region were malnourished, which is about 24.1 % of the entire population. This percentage was the highest, compared to other parts of the world (*42*). Undernutrition represents a double burden, with higher percentages of undernutrition and obesity. Both of these health hazards lead to diet-related non-communicable diseases. The occurrence of anaemia due to iron deficiency was around 23 % among children aged 1–3 in South Africa (*43*). Severe anaemia was responsible for more than 50 % of deaths among children with malaria in Africa. The prevalence of anaemia was around 54 % in a study conducted in Edo camp, Nigeria. Children aged 6-10 and those with malaria were more susceptible to anaemia. One in every two children in Nigeria suffers from iron deficiency (*44*, *45*). According to 2020 estimates, 21 % of the total population in Africa suffers from undernutrition. This shows the intensity of the disaster, which requires proper attention and prompt action from stakeholders, including citizens, health workers, food producers and processors, and government officials (*40*). The study emphasises the need for campaigns to educate the younger generation about the impact of food processing strategies and the components of a healthy diet.

Although the severity of the disease burden of undernutrition in developed countries is relatively low, it does exist and affects the population experiencing its various forms. In the USA, where food availability is not a concern, identifying undernourished population is possible by relying on social and economic indicators, rather than physiological indices (*46*). In Japan, malnourishment is a frequently occurring issue, the intensity of which increases with age. According to previous studies, undernourishment affects 1 to 10 % elderly people, although 41 to 48 % are deprived of proper nutrition (*47*-*49*).

The Government of India set up the National Nutrition Mission to address the challenges of undernourishment. This mission aims to compile the different nutrition-based schemes of government ministries and stakeholders, including the diversification of dietary patterns to enhance the intake of iron and folic acid and to encourage food fortification endeavours in the private sector (*36*). To combat anaemia, which is highly prevalent among the younger generation, anaemia prevention programs in schools, along with behaviour modification communication for modified diets and global iron supplementation, have been suggested as remedies for anaemia among school-going children (*38*). In Nepal, eleven policy documents highlighted nutrition interventions, including micronutrient supplementation, food fortification, feeding practices, and treatment of nutrient deficiency diseases (*50*). The provision of safe foods through a sustainable food system has been identified as a major policy area in Nepal (*50*). One cost-effective way to eradicate malnutrition among children in Africa is to provide a completely balanced meal for primary and secondary school children through school feeding programs, which has been implemented in many countries (*41*). It has also been recommended that national and international policies, along with nutritional intervention programs with the objective of prevention and treatment of undernutrition among the elderly, would be effective in Africa (*51*). Since the role of dietary modification in preventing disease conditions associated with malnutrition is evident from the above studies, the administration of functional foods is considered an ideal approach to eradicate malnutrition. Functional foods are known to fully satisfy the dietary requirements (*52*, *53*). Although only a few studies have established the connection between malnutrition and functional foods, the role of these foods in eliminating deficiency symptoms is highly evident (*54*).

The federal programs after the 1969 White House Conference on Food, Nutrition, and Health were found to be effective in the 1970s in diminishing the burden of undernourishment in the USA (*46*). Based on the information gathered from nutritional and dietary requirements, a “market basket” of inexpensive, frequently consumed foods that satisfy the Recommended Dietary Allowances (RDA) was created, which set the calculated minimum incomes by multiplying the price of an “RDA-based market basket” by the price of other needed commodities. This strategy could identify the undernourished population and enable more effective targeting of food programs (*46*). In Japan, a nutritional risk screening tool, SCREEN II (Seniors in the Community: Risk Evaluation for Eating and Nutrition, version II), a validated questionnaire with 14 items, is used effectively. This is intended to analyze the nutritional disorders in community-dwelling population above the age of 65. This tool accurately measures dietary habits, like food preparation, eating behaviour and food composition (*55*).

## THE ROLE OF FUNCTIONAL FOODS IN PREVENTING DEFICIENCY DISORDERS

Research interest in functional foods has gained momentum only in the early years of the 21^st^ century. This interest has a great impact on their market growth, which has shown a boom, and is anticipated to reach 280 billion US dollars by 2025, with an annual growth rate of 80 % (*56*). The definitions of functional foods have changed over time. In general, they are foods, either natural or processed, which contain bioactive ingredients and can provide potential health benefits apart from merely fulfilling satiety. Their therapeutic effects have been clinically proven (*57*). Some of these ingredients are antioxidants, probiotic organisms, essential fatty acids and polyphenols. The administration of foods fortified with iron has decreased the occurrence of anaemia (*58*). The water-soluble extract from tomato (marketed as Fruitflow) was the first product in Europe to gain an authorized, proprietary health claim under Article 13(5) of the European Health Claims Regulation 1924/2006 regarding nutrition and health claims made on foods (*59*). Effective antiplatelet components have been observed in Fruitflow, which strongly inhibited platelet aggregation and reduced blood pressure (*59*). Although the FDA does not have a clear-cut definition on functional foods, it has recently revised the definition of "healthy" foods, focusing more on whole food groups. The current revision aligns well with the present nutrition science and the Dietary Guidelines for the USA population. The latest rule, which came into effect in December 2024, permits nutrient-rich foods like fruits, vegetables and cereals to acquire "healthy" labels more easily (*60*).

### Plant-based functional foods

The bioavailability of iron has increased significantly in beverages fortified with more than one mineral. Additionally, enrichment with chickpeas, which are rich in iron and zinc, increased their iron and zinc content fivefold (*61*). Fortification with more than one nutrient increases the bioavailability of the added nutrients (*62*). An infant formula with sweet potato as the base was developed in Africa. This formula contained nutrients like vitamin A, iron and zinc, was of low cost and met CODEX standards for all micronutrients. However, it lacked calcium, which the author recommended to incorporate by adding dried powdered soft fish bones. The amount of iron in the formula was in the range of 8–10 mg/100 g, which was much higher than that in traditional weaning foods (*51*).

Meat analogues with nutritional content comparable to that of meat have been developed from plant sources for vegetarians and vegan consumers. To achieve this, microalgal powder and soy protein concentrate were effectively combined using high moisture extrusion technology. The resulting product had an appealing colour and a good nutritional profile, with high levels of nutrients like vitamins A and B. The fibrous texture preferred by consumers was achieved by adding 30 % microalgal powder (*63*).

Noodles were prepared by incorporating mushrooms, which can provide various health benefits, as they are known to be rich in protein and vitamin. They are also excellent sources of micronutrients like iron calcium, copper and zinc (*64*). They are low-calorie foods and are rich in dietary fibre. When mushrooms were incorporated at 5 %, sensory tests yielded good results. Additionally, the levels of protein, fibre, iron, calcium and potassium in the final product were reported to be higher than those in the noodle brands available on the market (*64*).

There are several naturally occurring plant-based polysaccharides like pectin, galactomannan, inulin, xanthan and gums, *etc.* (*65*). These polysaccharides have several applications in the food industry. It can be used as thickeners (*66*), stabilizers (*67*) and gelling agents (*68*). Konjac glucomannan was selenized by adding the trace element selenium. Through fortification, a new glucomannan hydrolase enzyme was formed that can convert konjac glucomannan to its corresponding oligosaccharides. Selenization was found to be beneficial for providing synergistic biological activity and for developing a functional ingredient. The newly developed konjac glucomannan oligosaccharides have antitumour activity (*69*).

The base material for most baked products is refined wheat flour. Although wheat flour provides energy and protein, it is deficient in minerals like calcium, iron and zinc (*70*). The popularity of bakery products like biscuits and cakes could be utilised to develop healthier variants. To enhance the bioaccessibility of minerals like calcium, iron and zinc and to produce a mineral-rich bread, wheat flour was replaced with sesame, cumin and moringa leaves in one combination, sesame and finger millet in another and sesame seeds in a third combination. All the bread samples developed had higher amounts of these minerals and the enrichment altered both the total and bioaccessible quantities of the minerals (*71*).

Wastewater from oil mills and olive paste, discarded from the olive oil industry, is rich in bioactive ingredients like polyphenols (*72*). These were added to enrich cereal foods like bread and pasta (*73*). After enrichment, it was observed that the products containing water from the oil mill had mildly improved chemical properties, while those enriched with olive paste showed a significant change in chemical properties. Additionally, products enriched with olive paste showed higher phenolic and antioxidant activity. Based on the overall quality index values of the products, it was reported that olive paste was more suitable for fortification than wastewater from oil mills (*73*).

### Dairy-based functional foods

Milk and milk products have been fortified with spices and herbs (*74*). Apart from providing flavour, herbs and spices offer protection against oxidative, inflammatory, hypertensive, diabetic and microbial infections, but create favourable conditions for the growth of beneficial microorganisms (*74*). The addition of cinnamon to yoghurt resulted in enhanced growth of lactic acid bacteria. The yoghurt also showed increased total phenolic content and radical scavenging activity (*74*). Milk from cow, buffalo and goat fortified with ginger and beetroot extracts showed improved antioxidant activity (*75*). New cottage cheese was prepared with the addition of spices like pepper, parsley, garlic, dill and rosemary (*76*). This cheese showed improved sensory properties, increased shelf life and higher biological value (*76*). Fortification of butter with rosemary herb reduced the rate of lipolysis in butter (*77*).

### Egg and meat-based functional foods

Table eggs were fortified with lipids from both microalgae and fish (*78*). The oil extracted from fish was rich in docosahexaenoic acid (DHA) and polyunsaturated fatty acids (PUFA). To retain the sensory properties of eggs, fish oil should be limited to 1.5 % (*78*). The sensory properties, acceptability and flavour of eggs supplemented with both oils were compared and it was observed that DHA supplemented with microalgal oil yielded better results in terms of sensory characteristics, overall acceptability and flavour of eggs (*79*).

Vegetable oils like sunflower oil in broiler chicken meat were replaced with PUFA (*80*). Soybean oil, mustard oil, fish oil and linseed oil were used as sources of PUFA. The meat was reported to be fortified with polyunsaturated fatty acids, with minimal variations in the sensory characteristics of the meat. The fortified meat showed enhanced levels of DHA and eicosapentaenoic acid (EPA) and lower levels of saturated fatty acids (*80*). Dietary recommendations for EPA and DHA based on cardiovascular risk considerations for European adults are between 250 and 500 mg/day (*81*). Supplemental intakes of DHA alone at 1 g/day do not raise safety concerns for the general population (*81*).

Broiler chicken meat was enriched by feeding chickens with high oleic peanuts, instead of grains, canola seeds and soybean meal (*82*). The protein content of peanuts is twice as high as that of grains. They are also rich in oleic acid (80 %). After enrichment, the meat showed increase amounts of polyunsaturated fatty acids and a low level of *trans*-elaidic acid, which when present in large amounts can lead to cardiovascular diseases. The protein and amino acid composition remained unaltered after enrichment (*82*).

When the feed of slow-growing chickens was enriched with linseed oil and tuna oil at 6 %, the chicken meat showed enhanced levels of PUFA with minimal alteration in the cholesterol content of the meat (*83*). The DHA content was observed to be 250 mg/100 g of meat, both in breast and thigh portions, which was adequate to meet the daily requirements of DHA, as per the European Food Safety Authority (EFSA) (*81*). According to EFSA, DHA is responsible for the proper functioning of the brain as well as vision (*81*).

Mono- and multilayered microcapsules were prepared from fish oil emulsions and added to meat products like sausages after cooking and curing (*84*). They served as vehicles for essential fatty acids, which are known for their beneficial effects on health. The capsules were developed from two combinations, one from lecithin and maltodextrin, and the other from lecithin and chitosan-maltodextrin. Through the addition of microcapsules, meat was fortified with EPA and DHA, and the products were analyzed to meet the labelling requirements for omega-3 fatty acid content according to the European Union legislation. The lipid profile of the product developed, as well as the percentage of fat loss, remained after fortification. The combination of lecithin and maltodextrin yielded better results than the other combinations in terms of bioavailability of EPA and DHA (*54*, *84*). Despite all the health benefits of fortified foods, they were seldom accepted by consumers wholeheartedly, primarily due to their unfamiliar taste and inconvenient processing methods (*85*). With the development of a sophisticated processing technology like 3D food printing, both the acceptability and accessibility of functional foods are expected to be enhanced to a greater extent.

## FABRICATION OF 3D-PRINTED PERSONALISED FUNCTIONAL FOODS

3D food printing can play a crucial role in addressing malnutrition. Since the technology can create foods that can offer personalized nutrition, 3D-printed foods meet the individual nutritional needs of customers. Although multinational brands like PepsiCo, Barilla, Mazola, Hershey's and Oreo have already launched their 3D products on the market (*86*), the literature highlights that recent research in 3D food printing should focus on developing new 3D-printed foods, with functional and bioactive components along with the basic nutrients (*87*). Table 3 (*11*, *27*-*30*, *88*-*91*) shows 3D-printed products fabricated from different ingredients.

**Table 3:** 3D-printed novel foods

Year	Category	Technology	Image	Nutritional goal	Target customer	Reference
2020	Egg-based	Extrusion	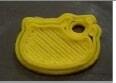	To understand whether eggs could be delivered in printable format. To compare the impact of printing technology on the yolk and egg white portions. To investigate whether the physical and chemical attributes of eggs could be employed effectively in layered manufacturing	All age groups	(*28*)
2020	Cereal-based	Extrusion	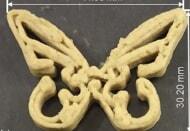	To fabricate healthier 3D food products from sustainable alternative raw materials. To develop a scientific base for 3D-printed mushroom-based products	All age groups	(*88*)
2018	Cereal-based	Extrusion	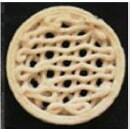	To construct novel foods containing functional ingredients. To study whether probiotic organisms could survive after baking in foods containing wheat flour.	All age groups	(*30*)
2021	Cereal-based	Extrusion	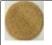	To investigate consumer acceptability of value-added food printed using processing waste. To ensure cleaner production procedures along with enhanced recovery of nutrients from industrial wastes. To describe newer and sustainable approach towards utilizing food processing wastes.	All age groups	(*89*)
2018	Fish-based	Extrusion	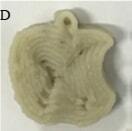	To investigate the possibility of fabricating 3D-printed surimi-based food To explore whether surimi could be printed in complex 3D patterns	All age groups	(*11*)
2019	Cereal-based	Extrusion	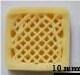	To investigate the influence of material composition on the quality of 3D-printed food. To explore the relationship between compressive pressure and needle velocity to achieve optimum parameters for 3D printing.	All age groups	(*90*)
2018	Milk-based	Extrusion	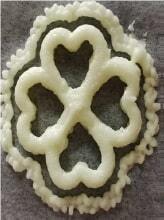	To construct protein-rich food simulants with 3D printing technique. To demonstrate 3D-printed simulant consisting of milk protein and to explore the impact of whey protein isolate incorporation on the printing behaviour of milk protein concentrate.	All age groups	(*29*)
2022	Black fungus-based	Extrusion	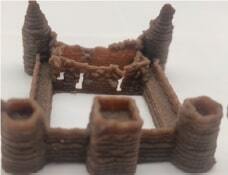	To develop 3D-printed dysphagia food with improved visual appeal and altered texture.	Age above 60 years	(*91*)
2018	Fruit-based	Extrusion	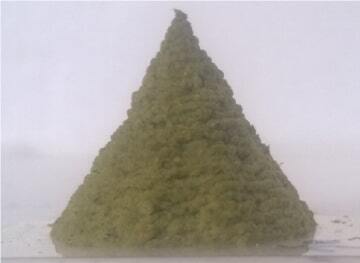	To explore the potential of a printing formulation consisting of fruits capable of providing energy, calcium, iron and vitamin D. To assess the sensory acceptability of foods with pyramid shape and their changes during storage.	Children in the age group of 3-10 years	(*27*)

### 3D food printing with food grade inks

Customized, highly porous, aqueous, 3D food simulants were developed with the food-grade pectin gel ink with low methylation (*92*). The incorporation of pectin resulted in a firmer product after printing. Products with different properties were printed by altering the composition of the food inks while keeping the printing parameters unchanged. Calcium chloride was added in amounts required to form cross-links of pectin, contributing to the 3D structure. Adding sugar resulted in a more viscous product with better printability and qualitative attributes. For printing formulations with a high pectin content, the amount of added calcium was reduced to achieve products with desired flow properties. Through this method, customized food inks with numerous applications could be developed to meet consumer preferences. That study may be considered an initial step in the fabrication of 3D-printed pectin-incorporated porous products (*92*).

A FujiFilm Dimatix inkjet printer using xanthan gum as the printing ink was employed with powdered cellulose (*93*). Xanthan gum was dispensed over the powdered cellulose for binding. properties and other parameters were optimized to improve the effectiveness of binding. Cellulose was selected as the binding agent due to its low cost and the ability to recrystallize after application. The newly formulated food-grade inks formed a firm structure with cellulose powder, which was used effectively for 2D inkjet printing. They could also be used successfully for 3D printing after recrystallization of the powdered cellulose (*93*). The performance of a 3D extruder-type printer at ambient temperature was assessed with model components such as modified starch and xanthan gum (*94*). The food inks used include carrot puree, xanthan gum pastes and modified starch. Rheological tests were performed and the results were analysed to evaluate whether the developed inks were suitable for printing. Carrot puree yielded better results for use as the food ink (*94*).

### 3D-printed foods with functional ingredients and antioxidants

A study was conducted to assess the relationship between the printability of formulations and their rheological properties using tomato paste as the model (*95*). It was observed that the pressure for extruding the material increased with higher flow stress. The dispensing behaviour of the material also increased proportionally with the flow stress, except for fat-containing products, which vary in their dispensing characteristics (*95*).

A 3D-printed lemon juice gel was developed by incorporating potato starch at different concentrations, and the influence of potato starch addition at different levels on various characteristics of the gel was studied (*96*). Lemon gel, which was otherwise less transparent and chewy, became printable with the incorporation of potato starch gel, which exhibited anti-ageing properties, transparency and the ability to retain moisture. The impact of printing parameters (nozzle height, nozzle diameter, extrusion rate and nozzle movement speed) as well as material properties on the quality of the final products was also investigated.

When potato starch was incorporated at 15 %, the flow and mechanical properties of the gel were observed to be optimum. The printing parameters were optimal when the nozzle diameter was around 1 mm, the extrusion rate was 24 mm/s and the nozzle movement speed was 30 mm/s. This work provided insight into the printing of gels in combination with starch (*96*). The impact of material composition on the quality of 3D-printed food was investigated using wheat flour, freeze-dried mango powder, olive oil and water (*90*). The optimal formulation with the best printing quality had flour and water in the ratio of 5:3 with no added freeze-dried mango powder and olive oil. For the formulation containing 2 % olive oil, the printing quality was optimal with the flour/water/olive oil ratio of 55:2.75:30. When 2.5 g mango powder was incorporated to the formulation, optimal printing quality was observed for the formulation with flour/water/olive oil/lyophilized mango powder ratio of 57.5:30:3:2.5. Additionally, a compressive pressure of 600 kPa, needle velocity of 6 mm/s, needle diameter of 0.58 mm and internal filling ratio of 60 % were found to be optimum. Foods printed with these printing parameters had numerous benefits like an organized packing structure, better interior texture profile and reduced deformation (*90*).

Mashed potatoes were printed three-dimensionally with different internal structures (*97*). The dimensional, structural and textural parameters were studied as a function of different infill patterns, infill percentages and shell perimeters. The results showed that the 3D-printed structures closely resembled the 3D designs greatly. Textural properties like hardness and gumminess were highly related to the infill levels. Microstructural analysis of the printed and cast materials revealed that the cast sample had a uniform internal structure, compared to the layered and porous structure of the printed material in longitudinal and cross- section, respectively. Textural analysis showed that the hardness of the printed sample was lower than that of the cast sample. That study demonstrated that 3D printing technology could alter the characteristics of foods, providing a novel approach for customizing the textural properties through the fabrication of internal structures (*97*).

A 3D-printed smoothie was developed from a mixture of fruit and vegetables of different colours (*27*). The ingredients used in the formulation were carrots, kiwifruit, avocado, pears and broccoli leaves. That study served as a stepping stone towards the printability of fruit- and vegetable-based formulations. The smoothie was printed in a pyramid shape and the 3D-printed version had a better visual appeal than its non-printed counterpart. The sensory properties, antioxidant and phenolic content were unaltered after printing. Two defects noted during printing were the discrepancy between the actual and estimated deposition positions of food and the inadequate sanitation of the printer areas in direct contact with food. The study explored the effect of printing variables like printing speed and flow on the printability of food containing fruit (*27*).

A snack food was formulated from fresh bananas, dried mushrooms, canned white beans, dried non-fat milk and lemon juice after 3D printing (*98*). The printed samples were similar to the 3D design. Material flow had a significant impact on the microstructural properties. When the material flow was reduced, the interior structure of the snacks was irregular with large pores, but when the flow was higher, the internal structure became thicker, with a decrease in porosity. The nutritional analysis of the printed snack revealed that 100 g of the snack was sufficient to meet the calorie requirements of children aged 3-10. The printed snack was reported to be rich in calcium, iron and vitamin D (*98*).

Another study explored the possibility of printing strawberry tree fruits, which have an exceptional nutritional profile, namely vitamins, antioxidants, minerals, sugars and bioactive components (*99*). Hydrocolloids like wheat flour and corn flour were added to the fruit blend at various amounts to make the formulation printable. The developed formulation was rich in polyphenols and had anti-microbial activity. The type and quantity of incorporated hydrocolloids, as well as the choice of printing program, played a significant role in the bioactive and functional properties of the formulation (*99*).

A Pickering emulsion rich in curcumin, initially stabilized by pea protein isolate and κ-carrageenan complex, was 3D printed (*100*). The printing results showed that the emulsion containing more than 0.3 % κ-carrageenan exhibited good extrusion, stability and shape retention. The bioaccessibility and stability of curcumin was greatly improved after *in vitro* digestion (*100*).

3D-printed biscuits were fabricated from processed flour and multi-grain flour, which contained cowpea sourdough and quinoa malt (*101*). Traditional biscuits were also developed and these two types of biscuits were compared in terms of colour profile, hardness and image parameters to examine the structural differences between the whole grain and composite flour (*101*). The 3D-printed biscuits were reported to be better due to their multi-flour formulation, low values for redness, browning index and hardness. Traditional biscuits were found to be harder than 3D-printed biscuits. The 3D-printed biscuits had similar quality characteristics to their non-printed counterparts. A slight reduction in antioxidant activity was observed after 3D printing, but the amount of minerals like iron and phosphorus was enhanced in 3D-printed biscuits (*101*).

### 3D-printed foods with cereals as the base material

3D-printed cereal foods containing probiotic microorganisms were produced from wheat dough by varying the amounts of water and calcium caseinate (*30*). The dough was printed in two designs: a honeycomb structure and a concentric circle. It was observed that the printability of the formulation depended on factors like water content, type of flour used and the quantity of additives like calcium caseinate. After printing, strains of probiotic bacteria were incorporated and the prints were baked. The results of the study revealed that the viability of probiotic bacteria increased with an increase in surface-to-volume ratio. Between the two structures, the honeycomb structure yielded higher number of viable bacteria than the other structure (*30*). The gel formation properties and the physical characteristics of the dough changed with any alteration in its composition. The study investigated the effect of these changes on 3D printing of the dough (*102*). The results showed that a pseudoplastic gel with high extrudability, gel strength, elasticity and low ductility was necessary to produce extruded samples of optimal shape. The formulation with water (29 g), sucrose (6.6 g), butter (6.0 g), flour (48 g) and egg (10.4 g) per 100 g yielded optimum results in terms of shape, gel formation and physical properties. Apart from optimizing the formulation for a good dough, the physical properties of all the dough formulations were studied. It was observed that sucrose, butter and flour were vital for the desired modelling results (*102*). A 3D pizza was developed from a gluten-free flour blend and its physical characteristics were compared with those of commercially available pizza (*103*). Although the fermentation time required for the gluten-free pizza formulation was double that required for commercial pizza dough, the textural properties and colour of the 3D-printed gluten-free pizza was comparable to those of the commercial pizza. The healthier version of pizza developed had the potential to replace commercially available pizza from wheat flour (*103*).

### 3D-printed foods for geriatric and dysphagic population

Around 50 % of the elderly population suffers from dysphagia, which is primarily characterized by difficulty in swallowing. To enable people with dysphagia to enjoy their meals, a 3D printer was developed to fabricate softer foods (*104*). A syringe pump was used to extrude the gel and a dispenser was employed to create 3D objects. Agar solution was used as the ink for printing. As agar gelation is influenced by temperature, the temperature of the gel should be regulated for optimal printing. Four types of soft foods were made using agar and gelatine with the developed printer. Compression tests and hardness measurements of the printed foods were carried out, and the results indicated that all the printed agar gels had a soft texture resembling jelly, which is appropriate for people with dysphagia (*104*). To meet the highly challenging requirements of dysphagic diet, novel edible formulations were developed from black fungus with the addition of gums like xanthan gum, gum Arabic and κ-carrageenan gum (*91*). It was reported that the formulation containing gum Arabic lacked self-supporting stability, and the results obtained for the International Dysphagia Diet Standardization Initiative (IDDSI) tests were poor (*91*). Although the formulation incorporated with carrageenan gum showed good self-supporting stability, the formulation failed the IDDSI tests due to its sticky nature, but the printed structure was self-stable. The formulation with xanthan gum closely resembled the level 5 minced and moist dysphagia food in the IDDSI tests (*91*). A dysphagia diet, suitable for the elderly population, was 3D printed (*105*). The diet was composed of cellulose nanocrystal-based (CNC) protein/polysaccharide edible inks, with protein sources including gelatine extracted from bovine skin and whey protein isolate, along with xanthan gum, an essential ingredient in dysphagia food. The formulation containing 2 % gelatine, 8 % whey protein isolate and 8 % xanthan gum was considered optimal due to its good printability and ability to retain shape. The formulation with bovine gelatine, xanthan gum and 1.5 % CNC met the criteria for level 5 minced and moist dysphagia diet according to the IDDSI guidelines (*105*).

The possibility of using flaxseed gum as the component with the ability to modify texture to develop dysphagia diets using 3D printing technology was studied (*106*). The ink formulation also contained mung bean protein and rose powder. The formulation containing 0.9 % flaxseed gum yielded optimal results in terms of printing characteristics and was classified a level 4 pureed/extremely thick dysphagia food according to the IDDSI guidelines. That study fabricated an inexpensive dysphagia diet containing phenolics exclusively from plant sources (*106*).

The impact of the incorporation of xanthan gum and guar gum on different properties of pork paste after cooking and 3D printing was investigated (*107*). The addition of hydrocolloids imparted a shear-thinning effect to the paste, improving the extrudability of the formulation. The incorporation of gums after the printing operations significantly altered the texture, making it suitable for people suffering from dysphagia. The final products could be classified as transitional foods after the IDDSI tests (*107*).

The 3D printer developed by Liu *et al.* (*108*) had a peristaltic pump and a pressurized tank instead of the syringe and piston commonly employed in other printers. This modification facilitated the printing of meat, which is otherwise complicated due to its fibrous structure. The benefits of the modified meat printer were improved storage capacity and material properties. Operational efficiency and productivity were increased. The chewing experience was enhanced after printing, facilitating easier intake by elderly people. This printing method could transfer ingredients from the interior of the meat to the surface (*108*).

### Protein- and fibre-enriched 3D-printed snack foods

3D-printed snack foods were fabricated from different ingredients like oat protein concentrate, faba bean protein, skimmed milk powder and starch (*109*). The study investigated whether extrusion-based 3D food printing could be used for pastes rich in starch, protein and fibre. The printability of the pastes was evaluated based on factors like the ease of extrusion, printing precision and print stability. The paste formulated with semi-skimmed milk powder achieved optimal printing precision and shape stability. The results showed that the applicability of the process depended entirely on the material and its binding properties. That study also demonstrated that the optimization of different formulations is a starting point for developing snack foods in future (*109*).

A 3D-printed snack food was developed from wheat flour fortified with ground larvae of yellow mealworms as a protein source (*110*). When insects were added at a rate of 20 %, a softer dough texture was obtained. The structural quality and nutritional characteristics were assessed and they remained unchanged after baking. To obtain a desirable product, baking was optimized at 22 min and 200 °C. The incorporation of insects improved the nutritional value in terms of protein quality and essential amino acid score (*110*).

A fibre-rich snack food was developed from button mushrooms using 3D printing technology (*88*). Lyophilized mushroom powder, which is not printable in nature, was made printable by incorporating different amounts of wheat flour. The formulation containing 20 % mushroom powder produced the best results in terms of nutritional value and printability. Sensory evaluation of the savoury products gave better results than the sweeter version of the snacks (*88*). To produce valuable by-products from industrial waste, functional cookies were developed using 3D printing technology from grape pomace and broken wheat, which would otherwise be discarded (*89*). An increase in grape content enhanced the viscosity of the formulation, which resulted in reduced printing speed. The developed cookie had a good sensory profile, structural integrity and was rich in protein and fibre (*89*).

Healthier 3D-printed snacks were made using composite flour from barnyard millet, green gram, fried gram and ajwain seeds (*111*). The printed products were subjected to different post-printing treatments. The printed snack had an attractive visual appeal and good overall acceptability. They also had protein and fibre in amounts needed to meet their daily requirements. Although the snacks developed using different post-processing methods received good sensory scores, microwave-dried snacks closely matched the untreated samples and showed better results in terms of nutritional and textural profile (*111*).

### 3D-printed foods from milk, eggs, meat and fish

A milk protein-based 3D food simulant was fabricated by incorporating whey protein isolate into milk protein concentrate (*29*). The optimal formulation had milk protein concentrate and whey protein isolate at a ratio of 5:2, demonstrating the applicability of a simulative high-protein system using extrusion technology. The prints based on this formulation closely resembled the digital model. The viscosity and mechanical strength of this formulation were adequate to facilitate deposition and adhesion, which are essential criteria in extrusion-based printing. That study would be useful to future works involving 3D printing of protein-rich foods (*29*).

The application of 3D printing to process commercially available cheese was investigated in another study (*112*). After printing, both printed and non-printed cheese were compared in terms of hardness and meltability. The printed cheese exhibited decreased hardness and increased meltability than the untreated cheese samples. The cheese samples after printing were found to be highly flexible in terms of geometry and texture. That study emphasized the role of 3D printing in altering the material properties, thereby constructing tailored products (*112*).

The study by Anukiruthika *et al*. (*28*) investigated the printability of both the white and yolk fractions of eggs. As eggs are highly nutritious and have numerous functional properties, 3D printing of egg-based formulations is highly relevant in the functional food industry. To make the egg fractions printable, rice flour was added as a filler at different proportions. Printing parameters including nozzle height, diameter, printing speed, extrusion motor speed and the rate of extrusion were optimized in the study. The incorporation of rice flour enhanced the strength and printing stability of egg whites and yolk. The printability of egg yolk was reported to be better than that of the egg white, with very little deformation. The optimum egg yolk formulation contained egg yolk and rice flour in a ratio of 1:2 (*28*).

Chicken meat was printed by incorporating composite millet as a source of dietary fibre (*113*). The composite millet-based flour comprised barnyard millet, fried gram, green gram and ajwain seeds. The optimal formulation contained chicken meat and flour in a ratio of 2:1. This ratio was selected based on the sensory results, as the incorporation of composite flour could adversely affect the sensory characteristics of the chicken meat. The printed product contained good amounts of both protein and dietary fibre, so that the product could be consumed on a daily basis by people of all the age groups. That study initiated the development of meat products enriched with dietary fibre, thus improving their health benefits (*113*).

Lean meat-lard composite layers were 3D printed and then cooked (*114*). It was reported that the structure of the products remained unaltered after cooking. The study investigated the effect of various fat content and infill densities on the physical and textural properties of meat. High fat content caused greater cooking loss, shrinkage, cohesiveness and lower fat retention, moisture retention, hardness and chewiness. With increased infill density, high moisture retention, and reduced shrinkage and cohesiveness, an increase in hardness and chewiness was observed (*114*). A 3D-printed fish surimi gel was developed and the effect of the addition of sodium chloride on the physiochemical properties of the gel, including water-holding capacity, gel strength and network structure was investigated (*11*). An increase in the addition of sodium chloride improved these properties and the gel containing 1.5 % sodium chloride yielded the best results in terms of rheological parameters. In that study, functional characteristics were the main criteria for printing surimi gel additively (*11*). Developing novel surimi products using fat fortification and 3D printing technology was also investigated. The work aimed to produce PUFA-rich high internal phase emulsions stabilized with microbial transglutaminase (MTGase) cross-linked fish scale gelatine (FSG) particles. Edible inks were developed by incorporating stabilized emulsion into surimi using 3D food printing and the effect of this addition on different characteristics of surimi gel was studied. When the oil phase volume was 80 %, the stability, flow properties and structural characteristics of the emulsion were affected by FSG. The mechanical properties of the emulsion-incorporated surimi were reported to be enhanced. The incorporation also had a significant effect on the printing accuracy. Incorporation of emulsions using 3D printing technology produces affordable, healthy, PUFA-enriched surimi products (*11*). Three-dimensional printing is now used to print different types of food components, through proper selection of raw materials, pre-printing steps to make them extrudable, optimization of formulations, and nozzle diameter and height. The ability to print multiple components, together with innovations in both software and hardware, enables this technology to fabricate foods for the general public, regardless of age group, occupation or lifestyle (*115*).

## CHALLENGES AND FUTURE PROSPECTS OF 3D FOOD PRINTING

Although 3D food printing has emerged as an invaluable technology, offering an array of possibilities in food processing, its limitations should not be overlooked. As this technology relies entirely on machines, with minimal dependence on human labour, variables like food formulation, extrusion motor force and the choice of raw materials for printing must be optimized prior to printing. The construction of 3D-printed food depends on temperature, nozzle dimensions, extrusion rate and deposition speed. Inadequate mixing of ingredients in the material feed, stability of the material feed during operation and post-printing factors often pose problems (*8*). Large-scale fabrication of foods is another limitation of 3D food printing due to lower production capacities. Additionally, these products have a very short shelf-life, since the formulations tend to be unstable owing to changes in the rheological properties (*8*). Manufacturing a reliable low-cost, large-scale printer capable of rapid prototyping remains a challenge (*116*). Furthermore, piracy of the 3D files may become an issue in the future as the technology gains popularity. The introduction of new policies under intellectual property rights to prevent copying of digital designs should not be overlooked in the future (*117*).

Food safety validation of customized formulations used in 3D food printing is necessary. The potential for food safety hazards upon consumption of 3D-printed foods must be taken into consideration. An important hazard arises from the interaction between the printing recipe and the parts of 3D printers, resulting in cross-contamination (*118*, *119*). Additionally, some printed foods require post-processing, which may involve heating and re-heating steps after storage. These processes can activate the growth of pathogenic organisms (*119*). Moreover, printed foods may contain components like allergens, foreign objects and ultrafine particles, which are hazardous to health (*27*, *119*, *120*). In future, to ensure safety of 3D-printed foods, regulatory standards addressing known risk factors and their controlling mechanisms associated with raw materials, printers, post-processing conditions and handling practices need to be emphasized (*27*, *119*). Dankar *et al*. (*121*) highlighted the importance of forming exclusive legislation for 3D food printing. Apart from the presence of hazardous substances and adulterants in 3D-printed foods, their consumption may lead to food poisoning outbreaks unless they are fabricated and stored properly. The regulation of these issues requires national, federal and international mandatory standards (*118*). In future, hypothetically, this technique may be used to fabricate novel foods from chemical components in the context of famine or food unavailability. In such scenarios, explicit regulation will be mandatory. 3D-printed foods could be categorized as imitation foods and should be labelled distinctly from their non-3D counterparts. If the production of traditional foods is more expensive than their 3D-printed versions and both these foods are indistinguishable, selling 3D-printed foods without distinct labelling would constitute food fraud (*122*). Even if most 3D-printed foods can be easily recognized due to their unique appearance, explicit labelling is highly recommended. A threshold (*e.g*. below a particular level) could be set as the labelling cut-oﬀ (*122*). Despite the shortcomings identified for this technique, 3D food printing has a promising future due to its ability to offer customization in every aspect of food. The technology contributes to environmentally friendly approach through efficient utilization of wastes and incorporation of sustainable resources. Providing viable dietary solutions in disasters and space nutrition are two major areas where this technology could be effectively applied in future (*123*).

## CONCLUSIONS

Three-dimensional (3D) printing of foods has emerged as a boon to the food processing sector since the technology is ideal for developing customized food products, in both geometry and nutritional profile. 3D food printing has immense potential to capture the future food market by utilising functional ingredients from around the world, whether from the surface, sub-surface or deep ocean, to provide healthier choices for consumers. Functional foods play a major role in the prevention and treatment of diseases associated with dietary disorders, as demonstrated by previous studies. Despite a highly health-conscious population, the functional food industry often fails to achieve consumer acceptance. The acceptability of these foods could be greatly enhanced through the fabrication of 3D-printed functional foods. Although 3D printing of foods is still in its early stages, studies on 3D-printed functional foods have shown that this technology can process a wide variety of ingredients, from lemon juice gel to highly fibrous meat, in countless designs, thus offering personalized nutrition. Despite the numerous challenges of operating a 3D printer, printed products are expected to oust the processed food market in the near future, considering the healthy and personalized options created in a short time.

## Data Availability

Data supporting this study are available from the respective authors upon reasonable request.
